# Computational fluid dynamics derived dataset for evaluation of mixing of a secondary solid phase in a circulating fluidized bed riser

**DOI:** 10.1016/j.dib.2023.109039

**Published:** 2023-03-08

**Authors:** Markku Nikku, Kari Myöhänen, Jouni Ritvanen, Timo Hyppänen

**Affiliations:** LUT University, School of Energy Systems, PL 20, FI-53851 Lappeenranta, Finland

**Keywords:** Fluidized bed, Fluidization, Fluctuation, Velocity, Volume fraction

## Abstract

Transient Eulerian simulations of multiphase flow inside a laboratory-scale circulating fluidized bed (CFB) riser were performed with air, bed material, and a secondary solid phase to evaluate the mixing of the secondary solid phase. This simulation data can be applied in model development or for computing terms that are commonly used when modeling mixing with simplified models (pseudo-steady state, non-convective models, etc.). The data was produced with transient Eulerian modeling using Ansys Fluent 19.2. The simulations were done with one fluidization velocity and bed material, while the density, particle size, and inlet velocity of the secondary solid phase was varied and 10 simulations per each secondary solid phase case were simulated for 1 s, each simulation having different starting conditions (flow state of the air and bed material) inside the riser. These 10 cases were then averaged to provide an average mixing profile for each secondary solid phase. Both the averaged and un-average data are included. The details of the modeling, averaging, geometry, materials, and cases are described in the open-access publication by Nikku et al. (Chem. Eng. Sci. 269, 118503).

**Specifications Table**

**Table:** 

Subject	Hydrodynamics, Chemical Engineering: Fluid Flow and Transfer Processes, Energy Engineering and Power Technology, Numerical Analysis.
Specific subject area	Fluidization, fluidized beds, mixing, computational fluid dynamics.
Type of data	Data file
How the data were acquired	Parallel processing on a Linux high-performance computing cluster using Ansys Fluent 19.2 was applied in transient Eulerian multiphase simulations with the geometry, mesh, and models described by Nikku et al. [1]. Pressure, velocity components, and volume fraction information of each phase (gas, bed material, and secondary solid phase) was exported for the whole geometry every 50 ms for each case. Multiple parallel cases were run, each with different initial conditions for the secondary solid phase feeding.
Data format	Raw Averaged
Description of data collection	The particle size, density, and inlet velocity of the secondary solid phase were varied to investigate the effect of each variable on the mixing of the secondary solid phase. The particle size of 50, 250, 500, 1000, and 5000 µm, material densities of 0.25, 0.625, 1.25, 1.875, 2.5, 3.125, and 5.0 g/cm³, and inlet velocities of 0.16, 0.64, 1.0, 2.0, 5.0 m/s were used for the secondary solid phase. The bed material particle size was 500 µm and the material density 2.5 g/cm³. Superficial fluidization velocity of air was 3.75 m/s, with density of 1.2 kg/m³ to present ambient conditions. To obtain a representative average for each case, it was found that 10 cases can be averaged [1], by taking an arithmetic average over 10 cases for each of the exported time steps for each individual computational cell. The averaging process was performed using Matlab R2019a on a desktop computer. The data for each time step and each initial condition was read from exported files into memory and then specified variables were added together in each cell and the sum was divided by the number of cases. The averaged data was written to a new data file for each time step (included in the data).
Data source location	• Institution: LUT University • City/Town/Region: Lappeenranta • Country: Finland • Latitude and longitude: 61.06599431210321, 28.094889464096088
Data accessibility	Repository name: Mendeley Data Data identification numbers: base cases - part 1: DOI:10.17632/n99mtzx33n.1 density cases - part 2: DOI:10.17632/dd9hy74wsx.1 size cases - part 3: DOI:10.17632/cggxvfxc49.1 velocity cases - part 4: DOI:10.17632/cvnn9mb5b5.1 Direct URLs to data: part 1: https://data.mendeley.com/datasets/n99mtzx33n/1 part 2: https://data.mendeley.com/datasets/dd9hy74wsx/1 part 3: https://data.mendeley.com/datasets/cggxvfxc49/1 part 4: https://data.mendeley.com/datasets/cvnn9mb5b5/1
Related research article	[1] Nikku, Markku; Myöhänen, Kari; Ritvanen, Jouni; Hyppänen, Timo (2023), Evaluation of mixing of a secondary solid phase in a circulating fluidized bed riser, Chemical Engineering Science 269, article 118503.

## Value of the Data

• There is a limited amount of data available on the mixing of solid phases inside circulating fluidized bed risers, as both experimental and numerical investigations are rare.
• The data is useful for understanding and studying mixing the phenomena inside circulating fluidized bed risers. It can be used, for example, to compute terms that are commonly used when modeling mixing with simplified models (pseudo-steady state, non-convective models, etc.), which can improve the modeling capabilities of these models.
• The data can be beneficial to fluidized bed researchers in academia as well as in the industry.
• The data can be useful for planning of similar experimental investigations, for example in probe/sensor placement and estimating the signal decay due to dense bed material concentrations.

## 1 Objective

The dataset was generated to study mixing process of a secondary, minor solid phase inside a circulating fluidized bed. These type of analyzes are extremely difficult to perform experimentally, thus research has mainly focused on using numerical methods, and limited number of these are available specifically in circulating fluidized bed conditions. Publication of the data adds value to the original manuscript by offering a vast amount of relatively detailed data available for all the researchers in the field to support their research. This in turn should reflect positively on the interest and impact of the original work.

## 2 Data Description

The data files are divided to four separate data sets (due to space limitations of the repository). The first part of the data set contains the 22 transient base cases as well as 10 and 22 case averaged versions (one .zip-file). The second part of the data set contains the 10 transient cases (each) for 0.25, 0.625, 1.25, 1.875, 3.125 and 5.0 g/cm³ as well as 10 case averaged version (each value in its own .zip-file). The third part of the data set contains the 10 transient cases (each) for 50, 250, 1000, and 5000 µm as well as 10 case averaged version (each value in its own .zip-file). The fourth of the data set contains the 10 transient cases (each) for 0.16, 0.64, 2.0, and 5.0 m/s as well as 10 case averaged version. (each value in its own .zip-file)

The previously mentioned cases correspond to those mentioned in Nikku et al. (2023) Table 2. The different cases are in their separate folders, labeled as Case1, Case2, etc. while the averaged folder contains the case averaged files. The ASCII data files have the format of Ansys Fluent interpolate files (.ip) and they are given with 50 ms intervals (labeled in the filename, for example file ending with 0450.ip corresponds with 450 ms after the beginning of the secondary solids feeding) for the whole riser and the data files contain: x-coordinate (perpendicular to feeding direction of the secondary solid phase), y-coordinate (vertical direction, gravity in -y direction), z-coordinate (parallel to feeding direction of the secondary solid phase), pressure, mp-1 (gas phase), mp-2 (bed material), mp-3 (secondary solid phase), x-velocity-1 (gas), y-velocity-1, z-velocity-1, x-velocity-2 (bed material), y-velocity-2, z-velocity-2, x-velocity-3 (secondary solid phase), y-velocity-3, z-velocity-3.

## 3 Experimental Design, Materials and Methods

The original research article [1] contains a detailed description of the simulation setup, section 2.3 and appendix 1, which can be followed to set up similar simulations in Ansys Fluent or similar software containing the capability to model multiphase flows with the kinetic theory of granular flows.

Three Eulerian phases are used with air as the primary phase, bed material as the secondary phase and the secondary solid phase as the tertiary phase. The gas phase is modeled as laminar, while the kinetic theory of granular flows is for the bed material and secondary solid phase as described in Appendix 1 of the original research article. The air properties are taken at ambient temperature (density equals 1.2 kg/m³ and dynamic viscosity equals 18·10^−6^ kg m^−1^ s^−1^) and using a fluidization velocity of 3.75 m/s at the inlet, while the bed material particle size is set to 500 µm and material density to 2500 kg/m³. For the solids return channel, a gas velocity of 0.5 m/s was implemented to reflect the fluidization air coming from the lift leg of the loopseal. For the bed material, a constant mass of 1.1 kg was maintained in the domain with an user-defined function which monitors the mass flow rate of bed material at the outlet, and implements the same mass flow rate as the inlet boundary condition at the solids return channel boundary.

The circulating fluidized bed flow fields are initialized by simulation the air and bed material flow until the circulation rate of bed material out of the riser is fluctuating around a relively stable average. The air-bed material two phase flow simulations are continued for 30 s, during which different starting conditions/times are gathered. After this, the mixing studies can be started. The mixing of the secondary solid phase is started at using different starting conditions/times that are arbitrarily chosen, but making sure there is atleast 250 ms difference between the starting states (to ensure possibility for the flow conditions to chance). As the starting conditions are quaranteed to affect to the mixing, the 22 simulations of the “base case” was simulated and later used to quantivy how many cases should be used in averaging to obtain relatively representative average of the mixing process in despite the different starting conditions. The secondary solid phase was introduced to the domain from a separate small inlet, and to monitor the mixing, the flow data (described in the section data description) was exported with 50 ms intervals. The mixing simulation latested for 1 s. The secondary solid phase properties were varied as mentioned previously to include a wide range of size and density ratios, as well as different inlet velocities for the secondary solid phase.

To account for the variation due to different starting conditions, a case averaging was performed using Matlab R2019a, where for each compational cell the value of quantity (for example volume fraction) is summed together with values from other similar cases and then divided with the number of cases, repeating this for each time step, as presented in equation (12) in the original manuscript. After obtain the average values for each computational cell in a time step, the averaged data was written in a separate data file (which are also included in the shared datasets). The averaging should be relatively simple to implement also in for example Python.

## Ethics Statements

The author assure that the work and manuscript follow and meet the ethical requirements of the journal. The work does not involve studies with humans or animals.

## CRediT authorship contribution statement

**Markku Nikku:** Investigation, Methodology, Software, Formal analysis, Writing – original draft, Writing – review & editing. **Kari Myöhänen:** Methodology, Software, Writing – review & editing. **Jouni Ritvanen:** Methodology, Conceptualization, Supervision. **Timo Hyppänen:** Supervision, Funding acquisition.

## Declaration of Competing Interest

The authors declare that they have no known competing financial interests or personal relationships that could have appeared to influence the work reported in this paper.

## Data Availability

Secondary solid phase mixing in a circulating fluidized bed - part 1: base case (Original data) (Mendeley Data). Secondary solid phase mixing in a circulating fluidized bed - part 2: density cases (Original data) (Mendeley Data). Secondary solid phase mixing in a circulating fluidized bed - part 3: size cases (Original data) (Mendeley Data). Secondary solid phase mixing in a circulating fluidized bed - part 4: velocity cases (Original data) (Mendeley Data).
